# Vascular Notch proteins and Notch signaling in the peri-implantation mouse uterus

**DOI:** 10.1186/s13221-015-0034-y

**Published:** 2015-12-01

**Authors:** Carrie J. Shawber, Lu Lin, Maria Gnarra, Mark V. Sauer, Virginia E. Papaioannou, Jan K. Kitajewski, Nataki C. Douglas

**Affiliations:** Department of Obstetrics and Gynecology, Division of Reproductive Sciences, College of Physicians and Surgeons, Columbia University Medical Center, 630 West 168th St, New York, NY 10032 USA; Department of Surgery, College of Physicians and Surgeons, Columbia University Medical Center, 630 West 168th St, New York, NY 10032 USA; Department of Obstetrics and Gynecology, Division of Reproductive Endocrinology and Infertility, College of Physicians and Surgeons, Columbia University Medical Center, 630 West 168th St, New York, NY 10032 USA; Department of Genetics and Development, College of Physicians and Surgeons, Columbia University Medical Center, 630 West 168th St, New York, NY 10032 USA; Department of Pathology and Cell Biology, College of Physicians and Surgeons, Columbia University Medical Center, 630 West 168th St, New York, NY 10032 USA

**Keywords:** Notch, Dll4, Jagged1, Endothelial cells, Pericytes, Decidua, Angiogenesis, Implantation

## Abstract

**Background:**

Angiogenesis is essential for uterine decidualization, the progesterone-mediated transformation of the uterus allowing embryo implantation and initiation of pregnancy. In the current study, we define the vasculature, expression of Notch proteins and Notch ligands, and Notch activity in both endothelial cells and vascular-associated mural cells of blood vessels in the pre-implantation endometrium and post-implantation decidua of the mouse uterus.

**Methods:**

We used immunofluorescence to determine the expression of Notch in endothelial cells and mural cells by co-staining for the endothelial cell marker, CD31, the pan-mural cell marker, platelet-derived growth factor receptor beta (PDGFR-β), the pericyte markers, neural/glial antigen 2 (NG2) and desmin, or the smooth muscle cell marker, alpha smooth muscle actin (SMA). A fluorescein isothiocyanate-labeled dextran tracer, was used to identify functional peri-implantation vasculature. *CBF:H2B-Venus* Notch reporter transgenic mice were used to determine Notch activity.

**Results:**

Notch signaling is observed in endothelial cells and pericytes in the peri-implantation uterus. Prior to implantation, Notch1, Notch2 and Notch4 and Notch ligand, Delta-like 4 (Dll4) are expressed in capillary endothelial cells, while Notch3 is expressed in the pericytes. Jagged1 is expressed in both capillary endothelial cells and pericytes. After implantation, Notch1, Notch4 and Dll4 are expressed in endothelial cells of newly formed decidual capillaries. Jagged1 is expressed in endothelial cells of spiral arteries and a subset of decidual pericytes. Notch proteins are not expressed in lymphatic vessels or macrophages in the peri-implantation uterus.

**Conclusions:**

We show Notch activity and distinct expression patterns for Notch proteins and ligands, suggesting unique roles for Notch1, Notch4, Dll4, and Jag1 during decidual angiogenesis and early placentation. These data set the stage for loss-of-function and gain-of-function studies that will determine the cell-type specific requirements for Notch proteins in decidual angiogenesis and placentation.

**Electronic supplementary material:**

The online version of this article (doi:10.1186/s13221-015-0034-y) contains supplementary material, which is available to authorized users.

## Introduction

Angiogenesis, the formation of new vessels from pre-existing vasculature, is critical in the uterine endometrium for embryo implantation, maintenance of early pregnancy, and development of the placenta. After fertilization, angiogenesis within the uterus occurs simultaneously with stromal cell decidualization, the rapid proliferation and differentiation of endometrial stromal fibroblasts into glycogen and lipid rich decidual cells [1–4]. In mice and humans, the uterine decidua supports early pregnancy prior to placenta development. The decidua serves as a scaffold for the newly formed decidual vascular plexus, as well as the maternal spiral arteries that are remodeled by embryo-derived trophoblasts during placenta formation. The decidual vascular plexus serves as the first exchange apparatus between the maternal circulation and the embryo and is necessary to maintain pregnancy prior to placenta formation [5–7]. In mice, inadequate decidual vascular development results in pregnancy failure by mid-gestation [7, 8]. In humans, inadequate decidual vascular development is associated with implantation failure, first trimester miscarriages, and abnormal placenta formation and function which leads to preeclampsia and intrauterine growth restriction [4, 9]. Ovarian estrogen and progesterone regulate decidua formation; however, the underlying molecular signaling pathways active in decidual angiogenesis have not as yet been fully characterized.

Sprouting angiogenesis is a multi-step process that begins with endothelial cells (ECs) sprouting out from mature vessels. ECs then migrate and proliferate to form a new sprout consisting of a tip cell at the front and neighboring stalk cells. New sprouts form capillary loops to create the vessel lumen and recruit vascular mural cells, which include pericytes and vascular smooth muscle cells (vSMCs), necessary for vessel stabilization [10]. Well-known regulators of angiogenesis during development and adult life include the vascular endothelial growth factor (VEGF) and Notch signaling pathways. In mice and non-human primates, VEGF activates VEGF receptors (VEGFR) to mediate increased uterine vascular permeability and decidual angiogenesis required for embryo implantation [5, 6, 11]. We have shown that inhibition of VEGFR-2, blocks decidual angiogenesis observed at embryonic day 7.5 (E7.5) and results in embryonic lethality prior to E10.5 [7]. Whereas continuous VEGFR-1 blockade significantly reduces decidual angiogenesis [12] and VEGFR-3 inhibition moderately reduces decidual angiogenesis [7], neither VEGFR-1 nor VEGFR-3 blockade has a notable negative effect on pregnancy prior to E10.5.

VEGF and Notch signaling pathways interact to coordinate developmental and postnatal angiogenesis [13–15], angiogenesis in tumors [16], and angiogenesis modeled *in vitro* [17–21]. Notch proteins (Notch1, Notch2, Notch3, and Notch4) are single-pass transmembrane receptors that interact with membrane-bound ligands of the Delta-like (Dll) (Dll1, Dll3, Dll4) and Jagged (Jag1 and Jag2) families in adjacent cells [22, 23]. In mice, Notch1 and Notch4 are expressed in endothelium of the developing vasculature [24–26] and Notch3 is expressed in mural cells, pericytes and vSMCs [26–28]. In tissues, such as the developing postnatal retina Notch ligand, Dll1 and Dll4 are expressed in ECs, while Jag1 is expressed in both ECs and vascular mural cells [23, 29]. Genetic studies demonstrate that Notch proteins and ligands are essential for embryonic vascular development [30–32] and maturation of vSMCs in mice [33, 34] and humans [35, 36].

Given the interactions between the Notch and VEGF signaling pathways in vascular development, Notch signaling likely functions in mammalian decidual angiogenesis to coordinate EC VEGFR signaling. A role for Dll4 in vascular development and differentiation in the decidua has recently been shown. Dll4 mediates decidual angiogenesis through induction of a tip/stalk phenotype in decidual ECs, suggesting a requirement for Notch signaling for proper decidual vascular development [37]. However, a comprehensive analysis of the expression of Notch proteins and ligands in decidual angiogenesis has yet to be described. The goal of this study is to define the expression of Notch proteins and Notch ligands in the peri-implantation uterus as a framework for genetic studies that will identify cell-type specific requirements for Notch signaling in decidual angiogenesis and placenta formation. Herein, we characterize the distribution of blood and lymphatic vessels, vascular associated mural cells, and macrophages in the pre- and post-implantation mouse uterus and use a fluorescein isothiocyanate (FITC)-labeled dextran tracer to identify the functional peri-implantation vasculature. We determine the expression of Notch proteins, Notch1-4, Notch ligands, Dll4 and Jag1, and Notch activity with respect to ECs and mural cells in the pre- and post-implantation mouse uterus. Our data provide strong support for a role for Notch signaling in decidual angiogenesis and pericyte/EC interactions.

## Methods

### Animals

The Columbia University Institutional Animal Care and Use Committee approved protocols used in animal studies. All mice were maintained on a C57BL/6 background. For assessment of wild type expression patterns, we used C57BL/6J virgin female mice and males of proven fertility (The Jackson Laboratory). The *CBF:H2B-Venus* transgenic mouse strain that expresses human histone H2B fused to yellow fluorescent protein (YFP) Venus in response to Notch/CSL transcriptional activation was used to determine Notch activity [38]. Mice were bred; noon on the day a mating plug was observed was designated embryonic day (E) 0.5. Pieces of uteri and implantation sites from pregnant females at E3.5 and E6.5, respectively, were embedded in Tissue-Tek® O.C.T.™ Compound (Sakura Fine Technical Co, Ltd, Tokyo, Japan), snap-frozen on dry ice in ethanol and stored at −80 °C in methylbutane (Sigma-Aldrich).

### Histology, immunohistochemistry (IHC) and immuofluorescence (IF)

7 μm transverse frozen sections through E3.5 uteri were generated. To visualize implantation sites at E6.5, 7 μm frozen sections through uteri/implantation sites, showing inter-embryonic regions and central parts of the decidua, were generated. Implantation was confirmed by hematoxylin and eosin (H&E) staining every 5th section. IHC and IF staining as previously described [39] was performed at least 3 times and 5 different uterine sections or implantation sites were analyzed for each antibody. Primary antibodies are listed in Table 1. The specificity of Notch protein and ligand primary antibodies was determined by comparing two independent antibodies for identical staining patterns in serial E9.75 and E10.5 tissue sections [40], as well as murine ovary sections. For colorimetric IHC, biotin goat anti-rat IgG (BD Biosciences 559286, 1:750), the avidin/biotin blocking kit (Vector SP-2001), the Vectastain ABC kit and DAB substrate kit (Vector SK-4100) were used. Sections were counterstained with hematoxylin. Secondary antibodies for IF are listed in Table 1. Vectashield containing with 4’, 6-diamidino-2-phenylindole (DAPI) (Vector H-1200) was used for nuclear visualization and mounting.

**Table 1:** Antibodies for analysis of the pregnant mouse uterus

Name	Host	Company	Catalog Number	Concentration
Primary antibodies				
CD11b	Rat	Abcam	ab8878	1:500
CD31	Rat	BD Biosciences	553370	1:350
Desmin	Rabbit	Abcam	ab15200	1:500
Dll4	Goat	R&D Systems	AF1389	1:100
F4/80	Rat	eBioscience	14-4801	1:100
Jagged1 extracellular domain	Goat	R&D Systems	AF599	1:100
Hey2	Rabbit	Abcam	ab25404	1:200
LYVE1	Rabbit	Abcam	ab14917	1:1000
NG2	Rabbit	Millipore	AB5320	1:750
Notch1	Goat	R&D Systems	AF1057	1:200
Notch2	Rabbit	Abcam	Ab8926	1:500
Notch3	Rabbit	Abcam	Ab60087	1:50
Notch4 Rb2-2	Rabbit	Dr. Kitajewski’s laboratory		1:100
PDGFR-β	Rabbit	Cell Signaling	3169	1:500
PDGFR-β	Rat	Abcam	Ab91066	1:100
α-SMA	Mouse	Sigma	C6198	1:850
Secondary antibodies				
Anti-goat-IgG Alexa-Fluor 594	Donkey	Invitrogen	A11058	1:800
Anti-rabbit-IgG Alexa-Fluor 594	Donkey	Invitrogen	A21207	1:800
Anti-rabbit-IgG Alexa-Fluor 488	Donkey	Invitrogen	A21206	1:800
Anti-rat-IgG Alexa-Fluor 594	Donkey	Invitrogen	A21209	1:800
Anti-rat-IgG Alexa-Fluor 488	Donkey	Invitrogen	A21208	1:800

### Dextran perfusion

Mice were given tail vein injections of 200 μl (25 mg/mL) of FITC-conjugated 40 kDa dextran (Invitrogen D-1820) at E3.5 and 10 kDa dextran (Invitrogen D-1845) at E6.5 [41–43]. After 10 minutes, animals were euthanized. Uteri and implantation sites were dissected in cold phosphate buffered saline, fixed in Carnoy’s solution and embedded in paraffin wax. Sections (7 μm) were deparaffinized, rehydrated and mounted in Vectashield medium containing DAPI or stained for Notch1 prior to mounting. Dextran was administered to 3 mice at each stage. Specific staining was performed at least 3 times and 5 different uterine sections or implantation sites were analyzed at each stage.

### Microscopy

IHC and H&E staining were examined with a Nikon MICROPHOT-FXA microscope and images were captured using NIS-Elements D3.10 software. Fluorescent images were captured using a Nikon A1 scanning confocal microscope on an Eclipse Ti microscope stand (Nikon Instruments, Melville, NY). Standard lasers and filters were used to image DAPI, AlexaFluor 488, and TRITC. Maximum intensity projections are shown.

## Results

### Characterization of blood vessels in the pre-implantation murine uterus

**Fig. 1:**
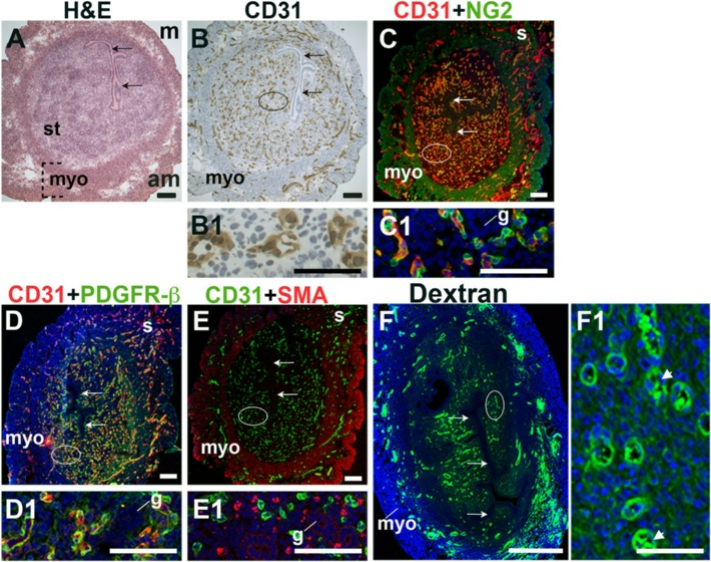
Characterization of blood vessels in the pre-implantation murine uterus. H&E, IHC, double staining IF and fluorescently labeled dextran in E3.5 transverse uterine sections. Ovals indicate areas of the uteri magnified (B1 – F1). The orientation is the same for all panels; anti-mesometrial (am) is at the bottom and mesometrial (m) is at the top. (A) H&E at E3.5 highlighting the luminal epithelium (arrows), inner circular and outer longitudinal myometrium [dashed bracket (myo)], and endometrial stroma (st). (B) CD31^+^ ECs are detected throughout the endometrial stroma and myometrium. (C) CD31 and NG2 staining. NG2^+^ mural cells are associated with CD31^+^ ECs in the stroma, myometrium and serosa (s). (D) CD31 and PDGFR-β staining. PDGFR-β^+^ murals cells are associated with CD31^+^ ECs in the stroma, myometrium and serosa. (E) CD31 and SMA staining. SMA labels glandular epithelium (g) and smooth muscle cells in the myometrium and serosa and is not associated with CD31^+^ ECs in the stroma. (F) FITC-dextran is detected throughout the endometrial stroma, in both small and large capillaries (F1, arrowheads), and myometrium. DAPI identifies all nuclei in IF images. Bar in A – E =100 μm. Bar in F = 500 μm. Bar in B1 – F1 = 50 μm.

Functional blood vessels, including capillaries, arterioles, and venules, consist of two key interacting cell types, ECs and vascular mural cells. ECs form the inner lining of the vessel wall and vascular mural cells, which include pericytes and vSMCs, surround the endothelial tube, providing structural support, regulating vessel diameter and aiding in the regulation of blood flow [44]. To characterize the pattern of vascular mural cells and functional blood vessels in the uterus before embryo implantation, we determined the distribution of ECs and mural cells by co-staining for the EC marker, CD31 and the pan-mural cell marker, platelet-derived growth factor receptor beta (PDGFR-β), the pericyte markers, neural/glial antigen 2 (NG2) and desmin, or the vSMC marker, alpha smooth muscle actin (SMA). Endometrial vessel functionality was determined by intravenous administration of FITC-labeled 40 kDa dextran into the tail vein of pregnant females followed by visualization of labeled dextran in uterine vessels, indicating vessel perfusion (Fig. 1).

At E3.5 prior to embryo implantation, CD31^+^ ECs in the endometrial stroma, myometrium and serosa are closely associated with NG2^+^ and PDGFR-β^+^ mural cells (Fig. 1C, D, yellow signal). Magnified areas of the endometrial stroma are representative of the stroma, myometrium and serosa (Fig. 1, C1, D1). Endometrial CD31^+^ vessels are not associated with SMA^+^ cells (Fig. 1E). SMA weakly labels glandular epithelia. SMA expression is seen throughout the myometrium and serosa, likely staining vSMCs, as well uterine smooth muscle cells (uSMCs) that also express NG2 and desmin (Fig. 1C, E, data not shown). Desmin expression is observed throughout the endometrium, stroma, myometrium and serosa and does not appear to be a specific marker of uterine vascular mural cells (data not shown).

The close association of NG2^+^, PDGFR-β^+^, and SMA^−^ pericytes with CD31^+^ ECs suggests that these vessels are functional capillaries. Intravenous injection of FITC-conjugated dextran shows dextran in vascular structures throughout the pre-implantation endometrial stroma (Fig. 1F). We conclude that functional capillaries are present a day prior to embryo implantation. These capillaries are evident as pericyte-covered CD31^+^ vessels within the stroma.

### Characterization of blood vessels in the post-implantation murine uterus

**Fig. 2:**
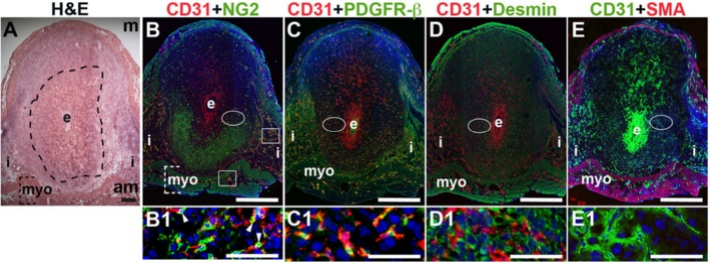
Characterization of endothelial cells and mural cells in the post-implantation uterus. H&E and double staining IF of E6.5 uterine sections through implantation sites, showing inter-embryonic regions and central parts of the decidua. Ovals indicate areas of the uteri magnified (B1 – E1). (A) H&E at E6.5 highlighting the embryo (e), inter-implantation sites (i), myometrium [dashed bracket (myo)], and decidua, which is between the embryo and dashed line. (B – E) CD31^+^ ECs are observed throughout the decidua, inter-implantation sites, and myometrium. (B) CD31 and NG2 staining. NG2^+^ mural cells are associated with CD31^+^ ECs in the myometrium (boxes) and in the decidua (arrowheads in B1). NG2^+^ mural cells are abundant in the anti-mesometrial (am) decidua and sparse in the mesometrial (m) decidua. (C) CD31 and PDGFR-β staining. PDGFR-β^+^ murals cells are associated with CD31^+^ ECs in the anti-mesomestrial decidua and myometrium. PDGFR-β ^+^ mural cells are abundant in the anti-mesometrial decidua and sparse in the mesometrial decidua (D) CD31 and desmin staining. Desmin^+^ cells are observed throughout the decidua and myometrium. (E) CD31 and SMA staining. SMA^+^ smooth muscle cells are observed in the myometrium and are not associated with CD31^+^ decidual ECs. DAPI identifies all nuclei in IF images. Bar in A = 100 μm. Bar in B – E = 500 μm. Bar in B1 – E1 = 50 μm.

At E6.5 after embryo implantation, the decidua is rich with CD31^+^ blood vessels necessary to maintain pregnancy prior to placentation that begins at E7.5 (Fig. 2B – E). In the myometrium and inter-implantation sites, NG2^+^ cells are closely associated with CD31^+^ ECs (Fig. 2B, boxes, yellow signal). In the decidua, NG2 expression is abundant in the anti-mesometrial region, but few NG2^+^ mural cells are associated with CD31^+^ ECs (Fig. 2B, arrowheads in B1). A majority of PDGFR-β^+^ cells are associated with CD31^+^ ECs with the heaviest coverage in the vessels in the anti-mesometrial decidua, myometrium and serosa (Fig. 2C, yellow signal). In contrast, very few NG2^+^ and PDGFR-β^+^ mural cells are found in the mesometrial decidua. Desmin expression is observed throughout the E6.5 implantation site, in decidual cells and stromal cells and in myometrium (Fig. 2D). Similar to E3.5, SMA labels vSMC and uSMCs in the E6.5 myometrium (Fig. 2E). Taken together, NG2^+^ and PDGFR-β^+^ cells associated with CD31^+^ ECs represent pericytes that likely have an active role in decidual angiogenesis.

**Fig. 3:**
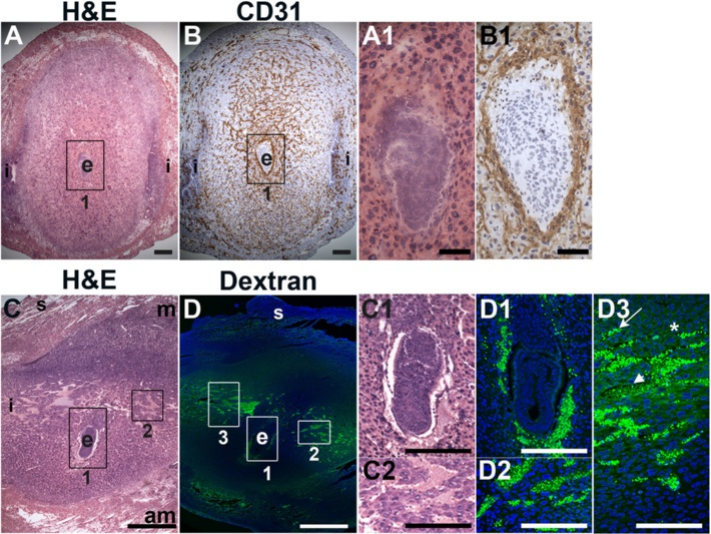
Distribution of endothelial cells and patent vessels identified by FITC-dextran in the post-implantation uterus. H&E, IHC and fluorescently labeled dextran in E6.5 uterine sections. (A) H&E at E6.5 highlighting the embryo (e) and inter-implantation sites (i). (B) CD31^+^ ECs are abundant in the decidua and myometrium. (C) H&E at E6.5 highlighting the embryo surrounded by blood filled maternal sinusoids (box 1, C1) and the blood filled lateral vascular sinuses in the mesometrial decidua (box 2, C2). (D) FITC-dextran is detected in capillaries (D3, arrow) and has extravasated into sinusoids around the embryo (D1), lateral vascular sinuses in the mesometrial decidua (D2 and D3, arrowhead), and the stroma (D3, asterisk). DAPI identifies all nuclei in IF images. am, anti-mesometrial; m, mesometrial; s, serosa. Bar in A and B = 100 μm. Bar in C and D = 500 μm. Bar in A1 and B1, C1, C2, D1 – D3 = 50 μm.

To determine the presence of functional decidual vessels, we performed FITC-conjugated 10 kDa dextran studies in E6.5 females. FITC-dextran identifies patent capillaries within the mesometrial decidua running laterally from the inter-implantation sites to the embryo (Fig. 3D, D2 and D3). These mesometrial vessels appear to be leaky as areas of extravasation are readily observed. FITC-dextran extravasation is seen into blood filled sinusoids in the implantation crypt around the embryo (Fig. 3D, D1), where CD31 staining is also present (Fig. 3D, B1), and in the lateral vascular sinuses (Fig. 3D, D2 and D3). The distribution of FITC-dextran suggests that functional, patent vessels as well as areas of increased vascular permeability are present in the mesometrial decidua, the site of placenta development.

### Characterization of lymphatic vessels and macrophages in the peri-implantation uterus

**Fig. 4:**
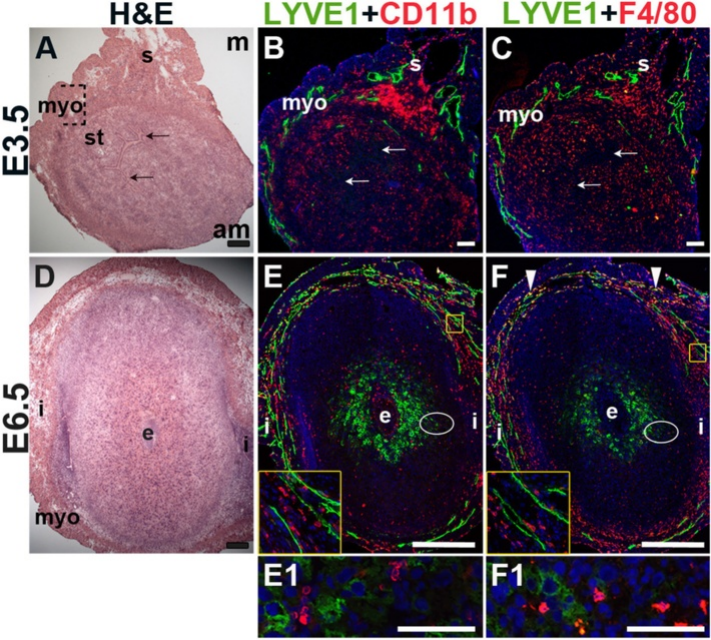
Characterization of lymphatic vessels and macrophages in the peri-implantation uterus. H&E and double staining IF of E3.5 (A – C) and E6.5 (D – F) uterine sections. Ovals indicate areas of the uteri magnified (E1 and F1). (A) H&E at E3.5 highlighting the luminal epithelium (arrows), inner circular and outer longitudinal myometrium [dashed bracket (myo)], endometrial stroma (st) and serosa (s). (B, C) LYVE1^+^ lymphatics are observed throughout the myometrium and serosa, but are not detected in the endometrial stroma. CD11b^+^ macrophages (B) and F4/80^+^ macrophages (C) are abundant throughout the endometrium, myometrium, and serosa, but their distribution patterns differ. (D) H&E at E6.5 highlighting the embryo (e), inter-implantation sites (i) and myometrium (myo). (E, F) LYVE1^+^ lymphatics are observed throughout the myometrium at E6.5. The LYVE1 positive staining in the decidua does not have a vascular pattern (E1, F1). CD11b^+^ macrophages (E) and F4/80^+^ macrophages (F) are abundant throughout the myometrium and serosa, but scattered throughout the decidua at E6.5. LYVE1^+^ F4/80^+^ macrophages are detected in the myometrium at E6.5 (F, white arrowheads). DAPI identifies all nuclei in IF images. am, anti-mesometrial; m-mesometrial. Bar in A – D =100 μm. Bar in E and F = 500 μm. Bar in E1 and F1 = 50 μm.

We used lymphatic endothelial hyaluronan receptor-1 (LYVE1) to detect lymphatic vessels [45] and CD11b [46, 47] and F4/80 [48–50] to detect macrophages. At E3.5, LYVE1^+^ lymphatic vessels are restricted to the myometrial and serosal layers of the uterus (Fig. 4B and C) and excluded from the endometrium and decidua. At E6.5, LYVE1^+^ cells are scattered throughout the decidua, without any apparent vascular pattern and likely do not represent lymphatic ECs (Fig. 4E, F), as LYVE1 is also expressed by a subset of macrophages [51]. F4/80 and LYVE1 double positive cells are observed in the myometrium at E6.5, but not at E3.5, suggesting that these cells are recruited subsequent to embryo implantation (Fig. 4F, arrowheads). The decidual LYVE1^+^ cells are neither CD11b, nor F4/80 positive indicating that they are not macrophages.

CD11b^+^ macrophages and F4/80^+^ macrophages are abundant throughout the myometrium and serosa at E3.5 and E6.5 (Fig. 4). These CD11b^+^ cells may be undifferentiated monocytes, as well as neutrophils or dendritic cells that also express this marker. Macrophages are abundant in the endometrial stroma before implantation (Fig. 4B, C), while their density is reduced throughout the decidua after implantation (Fig. 4E, F). The staining patterns suggest that different populations of macrophages exist within the peri-implantation uterus: a CD11b^+^ subset and a F4/80^+^ subset at both E3.5 and E6.5 and a LYVE1^+^ and F4/80^+^ double positive subset in the myometrium at E6.5.

### Notch1 expression in the peri-implantation uterus

**Fig. 5:**
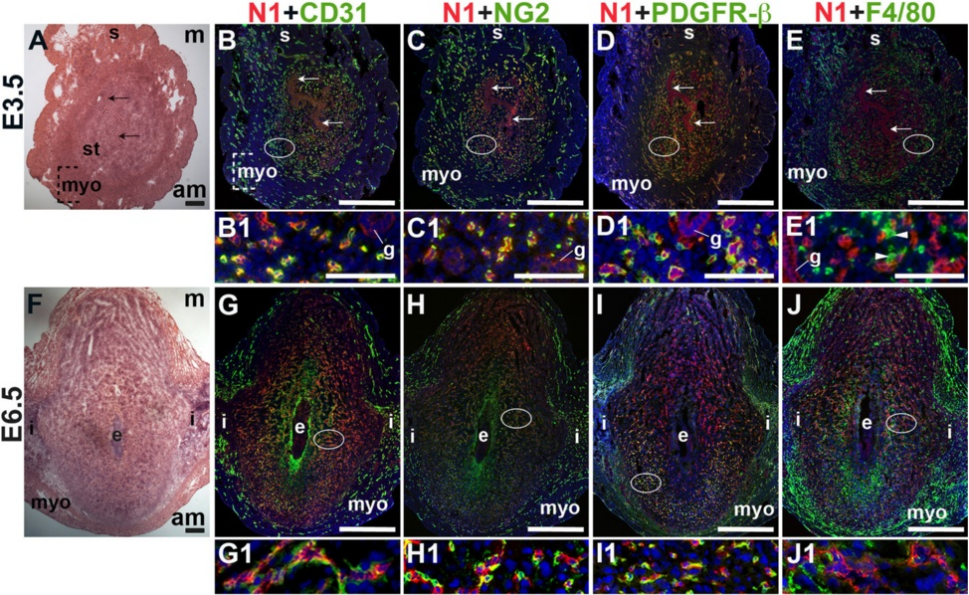
Notch1 (N1) expression in endothelial cells, mural cells and macrophages in the peri-implantation uterus. H&E and double staining IF of E3.5 (A – E) and E6.5 (F – J) uterine sections. Ovals indicate areas of the uteri magnified (B1 – E1 and G1 – J1). (A) H&E at E3.5 highlighting the luminal epithelium (arrows), inner circular and outer longitudinal myometrium [dashed bracket (myo)], endometrial stroma (st) and serosa (s). (B – E) Notch1^+^ cells are observed throughout the endometrial stroma, myometrium and serosa at E3.5. Low levels of Notch1 are detected in the luminal (arrows) and glandular epithelium (g). (B) Notch1 and CD31. Notch1 is expressed in CD31^+^ ECs. NG2^+^ mural cells (C) and PDGFR-β^+^ mural cells (D) are associated with Notch1^+^ cells in the stroma, myometrium, and serosa. (E) Notch1 and F4/80. F4/80 labels macrophages in stroma, myometrium and serosa. F4/80^+^ macrophages are adjacent to Notch1^+^ cells (arrowheads in E1). (F) H&E at E6.5 highlighting the embryo (e), inter-implantation sites (i), and myometrium (myo). (G – J) Notch1^+^ cells are observed throughout the decidua and inter-implantation sites at E6.5. (G) Notch1 and CD31. Notch1 is expressed in CD31^+^ decidual ECs. (H) Notch1 and NG2. NG2^+^ mural cells are associated with Notch1^+^ cells in the decidua. (I) Notch1 and PDFGR-β. PDGFR-β^+^ mural cells are associated with Notch1^+^ cells in the decidua. (J) Notch1 and F4/80. F4/80^+^ macrophages are not associated with Notch1^+^ cells in the decidua, myometrium and inter-implantation sites. DAPI identifies all nuclei in IF images. am, anti-mesometrial; m, mesometrial. Bar in A and F = 100 μm. Bar in B – E and G – J = 500 μm. Bar in B1 – E1 and G1 – J1 = 50 μm.

To define the expression of Notch1 in the peri-implantation uterus, we stained for Notch1 along with the EC marker, CD31, the pericyte markers, NG2 and PDGFR-β or the macrophage marker, F4/80. At E3.5, Notch1 is expressed predominantly in endometrial CD31^+^ ECs, while low levels of Notch1 are detected in luminal and glandular epithelial cells (Fig. 5A – D). At E6.5, Notch1 is expressed in decidual CD31^+^ ECs (Fig. 5G) of perfused vessels (Additional file 1: Figure S1). NG2^+^ and PDGFR-β^+^ pericytes are found in close association with Notch1^+^ ECs at both time points, suggesting that these pericytes cover Notch1^+^ ECs in capillaries (Fig. 5C, D, H, and I). At E6.5, NG2^+^ and PDGFR-β^+^ pericyte coverage of Notch1^+^ ECs is most complete in the anti-mesometrial decidua (Fig. 5H and I). F4/80^+^ macrophages are observed in between two Notch1^+^ ECs in the pre-implantation endometrial stroma (Fig. 5E, arrowheads). In the post-implantation decidua, F4/80^+^ macrophages are not associated with Notch1^+^ cells (Fig. 5F). In the peri-implantation uterus, Notch1 is expressed in the decidual capillary endothelium that is covered with NG2^+^ and PDGFR-β^+^ pericytes. Prior to implantation, these Notch1 expressing capillaries make contact with F4/80^+^ macrophages in the endometrium. Notch1 labels neither vSMCs, nor uSMCs in the myometrium and serosa.

### Notch4 expression in the peri-implantation uterus

**Fig. 6:**
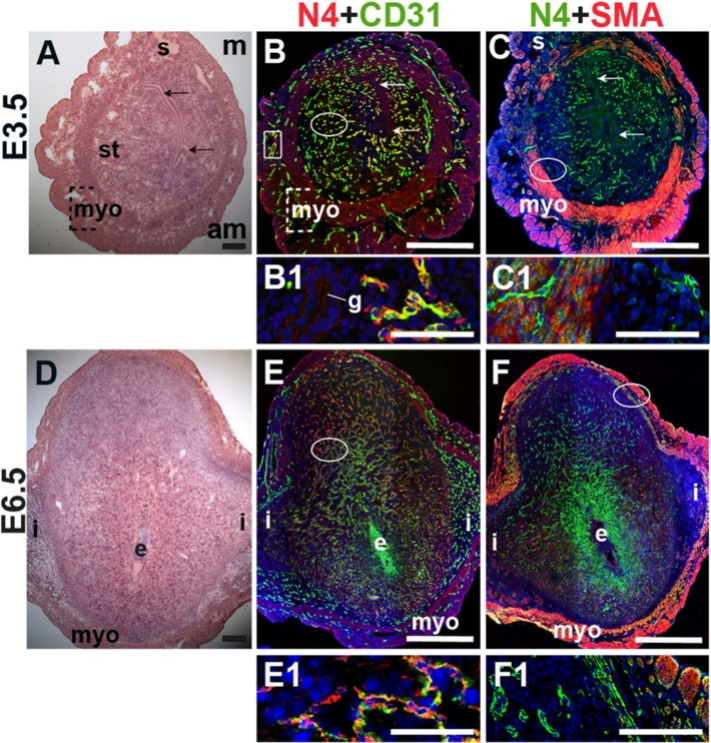
Notch4 (N4) expression in endothelial cells and mural cells in the peri-implantation uterus. H&E and double staining IF of E3.5 (A – C) and E6.5 (D – F) uterine sections. Ovals indicate areas of the uteri magnified (B1, C1, E1, F1). (A) H&E at E3.5 highlighting the luminal epithelium (arrows), inner circular and outer longitudinal myometrium [dashed bracket (myo)], endometrial stroma (st) and serosa (s). (B, C) Notch4^+^ cells are observed throughout the stroma, myometrium and serosa. Low levels of Notch4 are detected in the luminal (arrows) and glandular epithelium (g). (B) Notch4 and CD31. Notch4 is expressed in CD31^+^ ECs in the endometrium (oval) and myometrium (box). (C) Notch4 and SMA. SMA labels smooth muscle cells in the myometrium and serosa and is not associated with cells expressing Notch4. (D) H&E at E6.5 highlighting the embryo (e), inter-implantation sites (i), and myometrium (myo). (E, F) Notch4^+^ cells are observed throughout the decidua and myometrium at E6.5. (E) Notch4 and CD31. Notch4 is expressed in CD31^+^ ECs in the decidua. (F) Notch4 and SMA. Notch4 is not associated with SMA^+^ smooth muscle cells in the myometrium and serosa. DAPI identifies all nuclei in IF images. am, anti-mesometrial; m, mesometrial. Bar in A and D =100 μm. Bar in B, C, E, and F = 500 μm. Bar in B1, C1, E1, and F1 = 50 μm.

To define the expression of Notch4, we stained for Notch4 and the EC marker, CD31, or the SMC marker, SMA. At E3.5, Notch4 is expressed in CD31^+^ ECs throughout the endometrium and in a subset of large vessels in the myometrium (Fig. 6B, box highlights myometrial vessel). Notch4 is not detected in cells that express SMA in the myometrium and serosa (Fig. 6C and F). During post-implantation at E6.5, endothelial Notch4 expression is strongest in the mesometrial decidua where the placenta will form (Fig. 6E). Notch4^+^ cells are also observed in the myometrium and do not express SMA (Fig. 6F). Notch4 is mainly expressed in CD31^+^ ECs at E3.5 and in decidual ECs at E6.5. Our data show Notch4 expression in both capillaries and larger vessels in the peri-implantation uterus.

### Notch2 and Notch3 are predominantly expressed in SMCs in the peri-implantation uterus

**Fig. 7:**
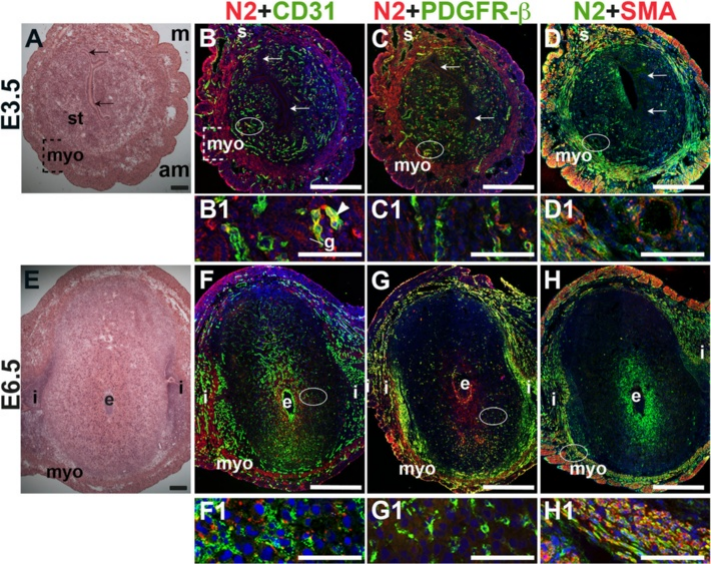
Location of Notch2 (N2) in relation to endothelial cells and mural cells in the peri-implantation uterus. H&E and double staining IF of E3.5 (A – D) and E6.5 (E – H) uterine sections. Ovals indicate areas of the uteri magnified (B1 – D1, F1 – H1). (A) H&E at E3.5 highlighting the luminal epithelium (arrows), and myometrium (myo), and endometrial stroma (st). (B – D) Notch2^+^ cells are observed throughout the stroma, myometrium and serosa (s), with low levels of Notch2 in endometrial glands (g). (B) Notch2 and CD31. Notch2^+^ cells in association with CD31^+^ ECs are scattered sparsely throughout the endometrium (arrrowhead in B1) and serosa (s). (C) Notch2 and PDGFR-β. Notch2 is not associated with PDGFR-β^+^ mural cells in the stroma and myometrium. (D) Notch2 and SMA. SMA^+^ smooth muscle cells are observed adjacent to Notch2^+^ cells in myometrium. Some uterine smooth muscle cells express both Notch2 and SMA. (E) H&E at E6.5 highlighting the embryo (e), inter-implantation sites (i), and myometrium (myo). (F – H) Notch2^+^ cells are observed throughout the myometrium. (F) Notch2 and CD31. Notch2 is not expressed in CD31^+^ decidual vessels. (G) Notch2 and PDGFR-β. Notch2 is not associated with PDGFR-β in the decidua or myometrium. (H) Notch2 and SMA staining. SMA^+^ smooth muscle cells are observed adjacent to Notch2^+^ cells in myometrium. Some uSMCs express both Notch2 and SMA. DAPI identifies all nuclei in IF images. am, anti-mesometrial; m, mesometrial. Bar in A and E =100 μm. Bar in B – D and F – H =500 μm. Bar in B1 – D1 and F1 – H1 = 50 μm.

To determine Notch2 and Notch3 expression patterns, E3.5 and E6.5 uteri were stained for Notch2 or Notch3 and EC marker, CD31 or mural cell markers, PDGFR-β and SMA. At E3.5, Notch2 expression is observed in a subset of CD31^+^ endometrial ECs (Fig. 7B, arrowhead). In contrast, Notch2 expression is not associated with CD31^+^ ECs in the E6.5 decidua (Fig. 7F). At both time points, Notch2 expression does not overlap with the vascular mural cell marker, PDGFR-β. In the myometrium, Notch2 is expressed in the SMA^+^ uSMCs of the inner circular and outer longitudinal fibers (Fig. 7D and H, yellow signal). The expression pattern of Notch2 in the decidua does not support a role for Notch2 in decidual angiogenesis.

**Fig. 8:**
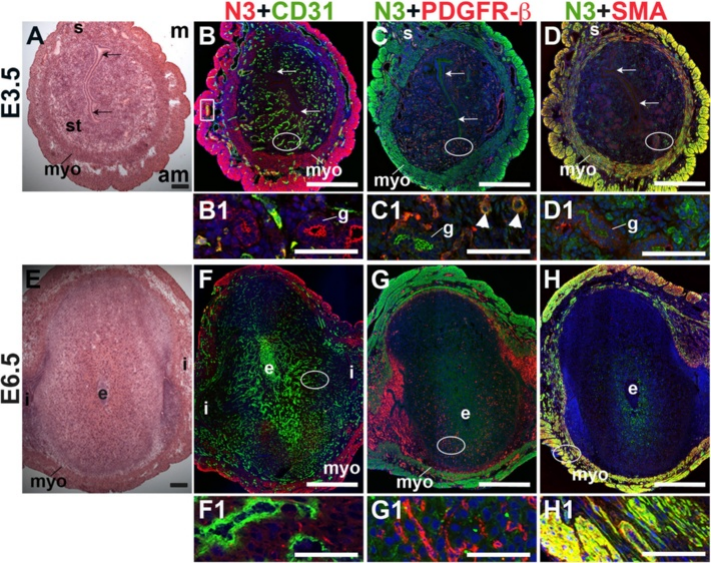
Location of Notch3 (N3) in relation to endothelial cells and mural cells in the peri-implantation uterus. H&E and double staining IF of E3.5 (A – D) and E6.5 (E – H) uterine sections. Ovals indicate areas of the uteri magnified (B1 – D1, F1 – H1). (A) H&E at E3.5 highlighting the luminal epithelium (arrows), inner circular and outer longitudinal myometrium [dashed bracket (myo)], endometrial stroma (st) and serosa (s). (B – D) Notch3^+^ cells are observed throughout the myometrium and serosa and in endometrial glands (g). (B) Notch3 and CD31. Notch3 is not expressed in CD31^+^ ECs. (C) Notch3 and PDGFR-β. Notch3 is expressed in PDGFR-β^+^ mural cells in the endometrial stroma (arrowheads in C1). (D) Notch3 and SMA. Notch3 and SMA staining overlaps in the myometrium and serosa. (E) H&E at E6.5 highlighting the embryo (e), inter-implantation sites (i), and myometrium (myo). (F – H) Notch3^+^ cells are observed throughout the myometrium. (F) Notch3 and CD31. Notch3 is not expressed in CD31^+^ ECs. (G) Notch3 and PDGFR-β. Notch3 is not associated with PDGFR-β in the decidua or myometrium. (H) Notch3 and SMA. Notch3 and SMA staining overlaps in the myometrium. DAPI identifies all nuclei in IF images. am, anti-mesometrial; m, mesometrial. Bar in A and E =100 μm. Bar in B – D and F – H =500 μm. Bar in B1 – D1 and F1 – H1 = 50 μm.

Notch3 is not expressed by CD31^+^ ECs in the peri-implantation murine uterus (Fig. 8B and F). In the E3.5 endometrium, Notch3 is expressed in PDGFR-β^+^ pericytes (arrowheads in C1) and in luminal and glandular epithelium, where it is localized apically (Fig. 8B – D). At both pre- and post-implantation time points, Notch3 is strongly expressed in uSMCs throughout the myometrium, as well as vSMCs surrounding large arteries (Fig. 8D and H). Notch3 is restricted to the vascular mural cells and myometrial SMCs in the peri-implantation murine uterus. As in other tissues where Notch3 has been examined [34, 52], Notch3 is a smooth muscle cell-specific protein in the pre- and post-implantation uterus.

### Angiogenic Notch ligand expression in the peri-implantation uterus

**Fig. 9:**
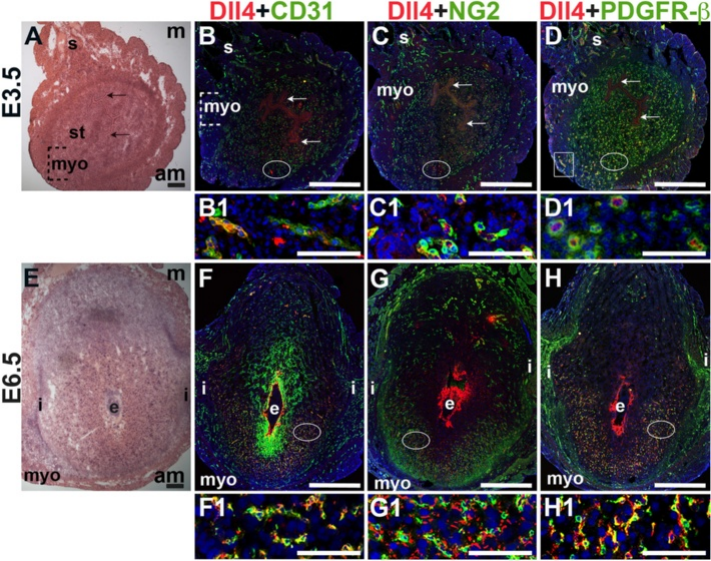
Expression of Notch ligand, Dll4 in relation to endothelial cells and mural cells in the peri-implantation uterus. H&E and double staining IF of E3.5 (A – D) and E6.5 (E – H) uterine sections. Ovals indicate areas of the uteri magnified (B1 – D1, F1 – H1). (A) H&E at E3.5 highlighting the luminal epithelium (arrows), inner circular and outer longitudinal myometrium [dashed bracket (myo)], endometrial stroma (st) and serosa (s). (B – D) Dll4^+^ cells are scattered throughout the stroma, myometrium (box) and serosa. (B) Dll4 and CD31. Dll4 is expressed in CD31^+^ ECs in the endometrial stroma. (C) Dll4 and NG2. NG2^+^ mural cells are observed adjacent to the Dll4^+^ cells in the stroma. (D) Dll4 and PDGFR-β. PDGFR-β^+^ mural cells surround Dll4^+^ stromal cells. (E) H&E at E6.5 highlighting the embryo (e), inter-implantation sites (i), and myometrium (myo). (F – H) At E6.5, Dll4^+^ cells are observed throughout the decidua, with most Dll4 expression in the anti-mesometrial decidua. (F) Dll4 and CD31. Dll4 is expressed in CD31^+^ decidual ECs. (G) Dll4 and NG2. NG2^+^ mural cells are adjacent to Dll4^+^ decidual cells in the anti-mesometrium. (H) Dll4 and PDGFR-β. PDGFR-β^+^ mural cells are adjacent to Dll4^+^ decidual cells in the anti-mesometrium. DAPI identifies all nuclei in IF images. Bar in A and E =100 μm. Bar in B – D and F – H =500 μm. Bar in B1 – D1 and F1 – H1 = 50 μm.

**Fig. 10:**
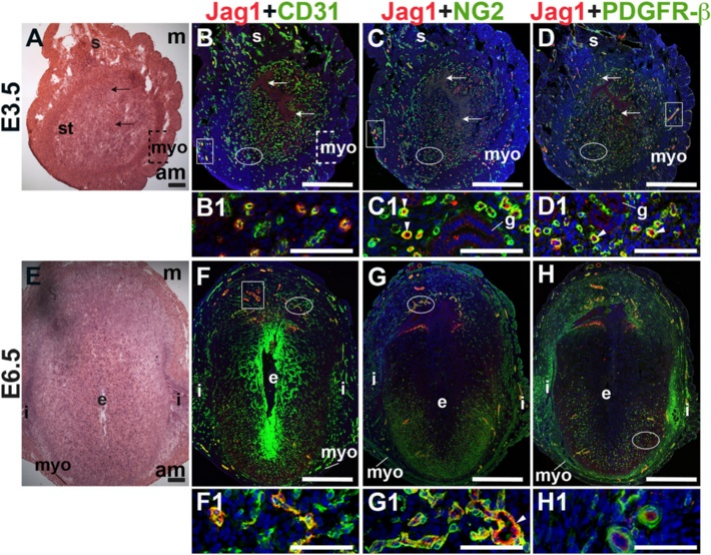
Expression of Notch ligand, Jagged1 (Jag1) in relation to endothelial cells and mural cells in the peri-implantation uterus. H&E and double staining IF of E3.5 (A – D) and E6.5 (E – H) uterine sections. Ovals indicate areas of the uteri magnified (B1 – D1, F1 – H1). (A) H&E at E3.5 highlighting the luminal epithelium (arrows), inner circular and outer longitudinal myometrium [dashed bracket (myo)], endometrial stroma (st) and serosa (s). (B – D) Jag1^+^ cells are observed throughout the endometrial stroma and in the myometrium (boxes). (B) Jag1 and CD31. Jag1 is expressed in CD31^+^ ECs in the stroma and myometrium. (C) Jag1 and NG2. Jag1 is expressed on a subset of NG2^+^ mural cells in the stroma (arrowheads in C1). (D) Jag1 and PDGFR-β. Jag1 is expressed on a subset of PDGFR-β^+^ mural cells in the stroma (arrowheads in D1). (E) H&E at E6.5 highlighting the embryo (e), inter-implantation sites (i), and myometrium (myo). (F – H) At E6.5, Jag1^+^ cells are observed in decidua and myometrium. (F) Jag1 and CD31. Jag1 is expressed in CD31^+^ ECs of spiral arteries (box) in the mesometrial decidua and in large vessels in the anti-mesometrial decidua and myometrium. (G) Jag1 and NG2 staining. Jag1 is expressed on a subset of NG2^+^ mural cells in the mesometrial decidua (arrowhead in G1). (H) Jag1 and PDGFR-β. PDGFR-β^+^ mural cells surround Jag1^+^ cells in the decidua. DAPI identifies all nuclei in IF images. am, anti-mesometrial; m, mesometrial. Bar in A and E =100 μm. Bar in B – D and F – H =500 μm. Bar in B1 – D1 and F1 – H1 = 50 μm.

E3.5 and E6.5 uteri were stained for the angiogenic Notch ligands, Dll4 and Jag1 and either the EC marker, CD31 or the pericyte markers, NG2 and PDGFR-β. In the E3.5 pre-implantation uterus, Dll4 and Jag1 are expressed in endometrial ECs (Figs. 9B and 10B). Dll4^+^ cells in the E3.5 endometrium are associated with NG2^+^ and PDGFR-β^+^ pericytes (Fig. 9C, D). Jag1^+^ is expressed on NG2^+^ and PDGFR-β^+^ pericytes (Fig. 10C, D, arrowheads highlight continuous yellow signal). In the E6.5 post-implantation decidua, most Dll4^+^ ECs are observed in the anti-mesometrial region (Fig. 9F). Whereas Dll4 is primarily expressed in small decidual capillaries, newly formed during decidual angiogenesis (Fig. 9), Jag1 is expressed in resident spiral arteries in the mesometrial decidua (Fig. 10F, box). At E6.5, NG2^+^ and PDGFR-β^+^ pericytes are still associated with Dll4^+^ and Jag1^+^ cells (Figs. 9G, H and 10G, H). The overlap of Jag1 and NG2 demonstrates that a subset of pericytes express Jag1 (Fig. 10G, arrowhead in G1). The different distribution of Jag1^+^ and Dll4^+^ cells suggests that Jag1 and Dll4 function in distinct populations of decidual ECs.

### Notch activity in ECs and pericytes in the peri-implantation uterus

**Fig. 11:**
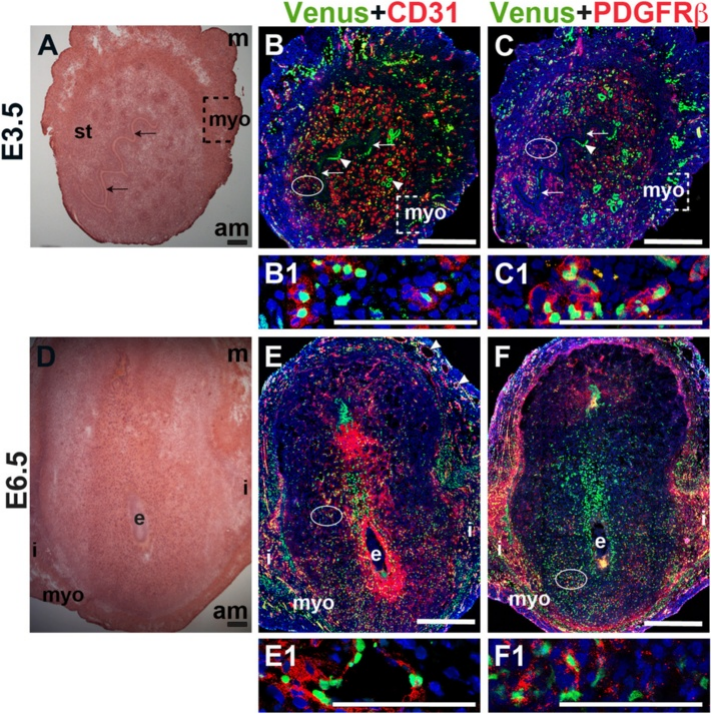
Notch activity in endothelial cells and mural cells in the peri-implantation uterus. (A) H&E at E3.5 highlighting the luminal epithelium (arrows), inner circular and outer longitudinal myometrium [dashed bracket (myo)] and endometrial stroma (st). (B, C) IF of sections through the uterus of *CBF:H2B-Venus* Notch reporter mice enabled assessment of Notch activity at E3.5. Ovals indicate areas of the uteri magnified (B1, C1). (B) Notch activity was observed in CD31^+^ ECs (red) in the stroma (B1), myometrium, and glandular epithelium (arrowheads). (C) Notch activity was observed in PDGFR-β^+^ mural cells in the stroma (C1) and myometrium. (D) H&E at E6.5 highlighting the embryo (e), inter-implantation sites (i), and myometrium (myo). (E, F) IF of sections through implantation sites of female *CBF:H2B-Venus* Notch reporter mice at E6.5. Ovals indicate areas of the uteri magnified (E1, F1). (E) Notch activity was observed in CD31^+^ ECs of decidual capillaries (E1) and myometrial vessels. (F) Notch activity was observed in PDGFR-β^+^ mural cells in the anti-mesometrial decidua. DAPI identifies all nuclei in IF images. am, anti-mesometrial; m, mesometrial. Bar in A and D =100 μm. Bar in B, C, E, and F = 500 μm. Bar in B1, C1, E1, and F1 = 100 μm.

To determine Notch activity in the peri-implantation uterus, female *CBF:H2B-Venus* Notch reporter mice were mated to wild-type males. YFP expression was used to determine Notch signaling activity in CD31^+^ decidual ECs and PDGFR-β^+^ pericytes. At E3.5, Notch activity is observed in CD31^+^ ECs in the endometrium and myometrium (Fig. 11B, B1), in luminal and glandular epithelial cells (Fig. 11B and C, arrowheads), and in a subset of PDGFR-β^+^ pericytes (Fig. 11C, C1). At E6.5, the *H2B-Venus* transgene is expressed in CD31^+^ ECs of decidual capillaries (Fig. 11E, E1), CD31^+^ ECs of myometrial vessels (Fig. 11E) and in a subset of PDGFR-β^+^ pericytes in the anti-mesometrial decidua (Fig. 11F, F1). Thus, Notch activity is observed in ECs and pericytes in the peri-implantation uterus.

**Fig. 12:**
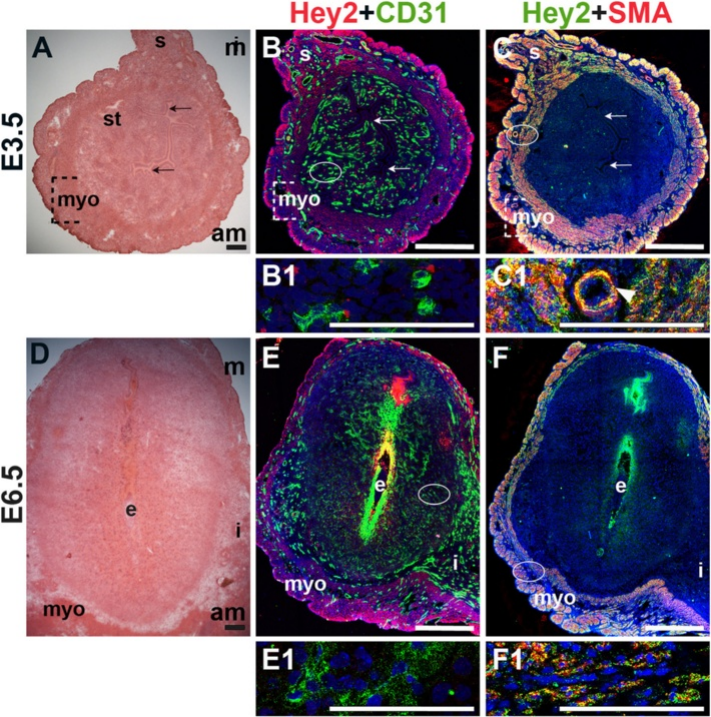
Expression of Notch effector protein, Hey2, in relation to endothelial cells and mural cells in the peri-implantation uterus. H&E and double staining IF of E3.5 (A – C) and E6.5 (D – F) uterine sections. Ovals indicate areas of the uteri magnified (B1, C1, E1 and F1). (A) H&E at E3.5 highlighting the luminal epithelium (arrows), inner circular and outer longitudinal myometrium [dashed bracket (myo)], endometrial stroma (st) and serosa (s). (B, C) Hey2^+^ cells are observed throughout the myometrium and serosa. (B) Hey2 and CD31. Hey2 is not expressed in CD31^+^ ECs (B1). (C) Hey2 and SMA. Hey2 and SMA staining overlaps in the myometrium and serosa (C1). (D) H&E at E6.5 highlighting the embryo (e), inter-implantation sites (i), and myometrium (myo). (E, F) Hey2^+^ cells are observed throughout the myometrium. (E) Hey2 and CD31. Hey2 is not expressed in CD31^+^ ECs of decidual capillaries (E1). (F) Hey2 and SMA. Hey2 and SMA staining overlaps in the myometrium (F1). DAPI identifies all nuclei in IF images. am, anti-mesometrial; m, mesometrial. Bar in A and D =100 μm. Bar in B, C, E, and F =500 μm. Bar in B1, C1, E1 and F1 = 100 μm.

We assessed the expression of Hey2, a downstream effector of Notch signaling, in CD31^+^ ECs and SMA^+^ SMCs in the peri-implantation uterus. Hey2 is not expressed by CD31^+^ ECs in the peri-implantation murine uterus (Fig. 12B and E). At both pre- and post-implantation time points, Hey2 is strongly expressed in uSMCs throughout the myometrium (Fig. 12C and F), as well as vSMCs surrounding large arteries (Fig. 12C, C1, arrowhead).

## Discussion

Transformation of the pre-implantation E3.5 uterine endometrium to the post-implantation E6.5 uterine decidua requires angiogenesis and vascular remodeling to increase vascular permeability, and immune/inflammatory changes. This vascularization process assures successful embryo implantation and placenta formation [1, 2]. Herein we show that prior to implantation, most vessels in the uterine endometrium are capillaries, defined as CD31^+^ ECs covered by NG2^+^/PDGFR-β^+^ pericytes and lacking SMA expression. Macrophages are abundant throughout the endometrium, myometrium and serosa, whereas lymphatic vessels are restricted to the myometrium and serosa. After implantation, we found patent vessels lateral to the embryo running between the inter-implantation sites and the embryo. NG2^+^ and PDGFR-β^+^ pericytes are most abundant on newly formed decidual capillaries in the anti-mesometrial decidua. It is plausible that vessels in the mesometrial decidua, the site of vascular remodeling during placenta formation, may not require extensive pericyte coverage. Alternatively, a different population of pericytes, that does not express NG2, but may express desmin, could support the mesometrial vessels that do not express NG2. However, desmin is also expressed in the stromal cells and decidual cells and thus it is not possible to discriminate between desmin^+^ pericytes and stromal cells.

We hypothesized that Notch proteins and ligands would be differentially expressed in the vasculature and vascular associated mural cells of the pre-implantation uterus and post-implantation uterine decidua, reflecting their possible functions during decidualization. Prior to implantation, Notch1, Notch2 and Notch4 are expressed in the ECs and Notch3 is expressed in pericytes (Table 2). Notch1 EC expression is observed in the perfused capillaries containing FITC-labeled dextran. The Notch ligands, Dll4 and Jag1 are expressed in a subset of ECs, while Jag1 expression is observed in a subset of pericytes (Table 2). These Notch proteins and ligands are thus associated with pre-implantation endometrial capillaries. After implantation, we find Notch1, Notch4, Dll4, and Jag1 expression in decidual ECs and Jag1 expression in a subset of pericytes (Table 2). We observe EC Dll4 in the angiogenic vessels of the decidua (Table 2), consistent with the role of Dll4 in newly forming sprouts during angiogenesis [37]. Notch2, Notch3 and Notch4 are expressed in the peri-implantation myometrium. These data provide an essential foundation for conducting mechanistic studies to define the roles of individual Notch family members in decidual angiogenesis and placentation.

**Table 2:** Summary of Notch activity and expression in the peri-implantation mouse uterus

		Notch signaling	Notch1	Notch2	Notch3	Notch4	Dll4	Jag1
E3.5 Pre-implantation								
	Endothelial cells	X	X	X		X	X	X
	Pericytes	X			X			X
	vSMCs	X			X			
	uSMCs	X		X	X			
E6.5 Post-implantation								
	Endothelial cells	X	X			X	X	X
	Pericytes	X						X
	vSMCs	X			X			
	uSMCs	X		X	X			

uSMCs, uterine smooth muscle cells; vSMCs, vascular smooth muscle cells

*Notch1* and *Notch1/Notch4* mutant embryos have lethal angiogenic defects, with more severe phenotypes in *Notch1/Notch4* double mutants [30]. Endothelium-specific activation of Notch1 or Notch4 and disruption of Notch signaling within the vasculature [24, 31, 32] also results in embryonic lethality by mid-gestation with defects in fetal angiogenesis. These mutants reveal the importance of Notch1 and Notch4 in formation of fetal-derived embryonic and placental components. Notch1 has been previously been reported to be expressed in mouse and primate maternal uterine stromal cells during decidualization [53, 54]. The contribution of Notch1 and Notch4 signaling to maternal-derived placental vascular components or the pre-placental decidual vasculature has not as yet been investigated. We show that Notch1 is expressed in capillaries and excluded from macrophages in the peri-implantation uterus. Notch1^+^ ECs are uniformly distributed throughout the decidua, while Notch4 expression in ECs is more abundant in the mesometrial decidua. The partially overlapping expression patterns for Notch1 and Notch4 suggests that Notch1 and Notch4 may have some functionally redundancy in decidual angiogenesis. Further, active Notch signaling in both decidual ECs, which express Notch1 and Notch4, and pericytes suggests that the Notch pathway has a fundamental role in decidual angiogenesis.

Homozygous null mutants of *Jag1* and *Dll4* die during embryogenesis due to vascular defects [26, 55–58]. The umbilical artery and placental blood vessels are decreased in size in *Dll4* heterozygous mutant embryos [59]. The requirement for Dll4-mediated signaling in decidual angiogenesis was recently investigated [37]. Peri-implantation administration of a Dll4 blocking antibody resulted in increased, but non-productive decidual angiogenesis that compromised pregnancies by E9.5, with very few embryos surviving to E13.5 [37]. In this model, Dll4-mediated signaling was blocked during decidualization, however it is unclear whether subsequent placentation was affected, leading to the abnormal embryonic development observed. Our finding of Dll4 in capillaries in the anti-mesometrial uterine decidua supports the finding that Dll4 signaling is required for proper decidual angiogenesis. Jag1 is expressed in decidual ECs of maternal spiral arteries and in pericytes. The different expression patterns of Dll4 and Jag1 in the decidual vasculature suggest unique functions for these Notch ligands.

During pregnancy, the lymphatic vasculature is believed to play a role in regulating the fluid balance between the maternal and fetal compartments and in maintaining maternal tolerance of the semi-allogeneic fetus [45, 60, 61]. There is general agreement that the myometrium and serosa of both humans and mice contain lymphatic vessels [60, 62, 63]. Whereas LYVE1^+^ lymphatics have not been detected in the endometrium of the non-pregnant human uterus [60, 64], podoplanin^+^ lymphatics are abundant in the endometrium basalis, the region directly adjacent to the myometrium and sparse in the endometrium functionalis, the region that is shed during menses [63]. LYVE1^+^ lymphatics are prominent in the decidua during all trimesters of human pregnancy, but have not been detected in the murine decidua [60]. Similarly, we do not observe LYVE1^+^ lymphatic vessels in the peri-implantation murine decidua. Further investigation with additional lymphatic markers is necessary to confirm the difference between humans and mice.

Pregnancy is associated with an influx of macrophages into the uterus [65, 66]. Macrophages are proposed to function in coordinating the maternal immune response, in apoptosis and tissue remodeling at the maternal-fetal interface, as well as in promoting angiogenesis [67, 68]. Macrophages are recruited into the endometrium during the peri-implantation period such that decidual macrophages are the second most abundant immune cell population at the implantation site, comprising 20-30 % of immune cells in the uterine decidua [69, 70]. Whereas CD11b is expressed by myeloid cells other than macrophages, F4/80 is expressed by mature macrophages [46, 50]. F4/80 is commonly used to identify macrophages in the female reproductive tract [12, 71–73]. We recently showed that VEGFR-1^+^ ECs are often in direct contact with CD11b^+^ monocytes/macrophages and F4/80^+^ macrophages in the peri-implantation uterus [12]. Blockade of VEGFR-1 significantly decreases both the number macrophages in the decidua of E7.5 pregnant mice, likely as an indirect result of the decrease in VEGFR-1 function in ECs [12]. Here we show F4/80^+^ macrophages in direct contact with Notch1^+^ vessels prior to implantation. Available data suggest that macrophages do not have an obligatory role in uterine decidualization and embryo implantation [12, 71]. However, macrophages are implicated in trophoblast invasion and spiral artery remodeling during placental development [74, 75].

## Conclusions

Taken together, our findings support unique roles for Notch1, Notch4 and angiogenic ligands, Dll4 and Jag1 in decidual angiogenesis. Abnormal decidual angiogenesis can result in spontaneous miscarriages early in pregnancy or have adverse “ripple effects” throughout pregnancy, leading to abnormal placentation, fetal growth restriction, and/or preeclampsia [1]. Proper trophoblast invasion of maternal spiral arteries in the uterine decidua is integral to placenta formation. Notch proteins are implicated in EC-trophoblast interactions during this vascular remodeling in the uterine decidua [76]. Our data set the stage for genetic studies to evaluate the requirement for Notch signaling in decidual angiogenesis and early placentation.

## Additional files

Additional file 1: Figure S1. Notch1 expression in patent capillaries in the pre-implantation uterus at E3.5. Notch1 expression is detected in capillaries containing FITC-dextran. The area in the rectangle is magnified and shown in panels on the right. DAPI identifies all nuclei. Scale bar = 50 μm. (JPG 373 kb)

## Abbreviations

ECs: endothelial cells
Dll4: Delta-like 4
Jag1: Jagged 1
NG2: neural/glial antigen 2
PDGFR-β: platelet-derived growth factor receptor beta
SMA: alpha smooth muscle actin
uSMCs: uterine smooth muscle cells
vSMCs: vascular smooth muscle cells
VEGFR: vascular endothelial growth factor receptor
YFP: yellow fluorescent protein
