# Supplementary figures and images for: Vascular Notch proteins and Notch signaling in the peri-implantation mouse uterus
Source: Vasc Cell. 2015 Dec 1;7:9. doi: 10.1186/s13221-015-0034-y (PMC4666149; doi:10.1186/s13221-015-0034-y)

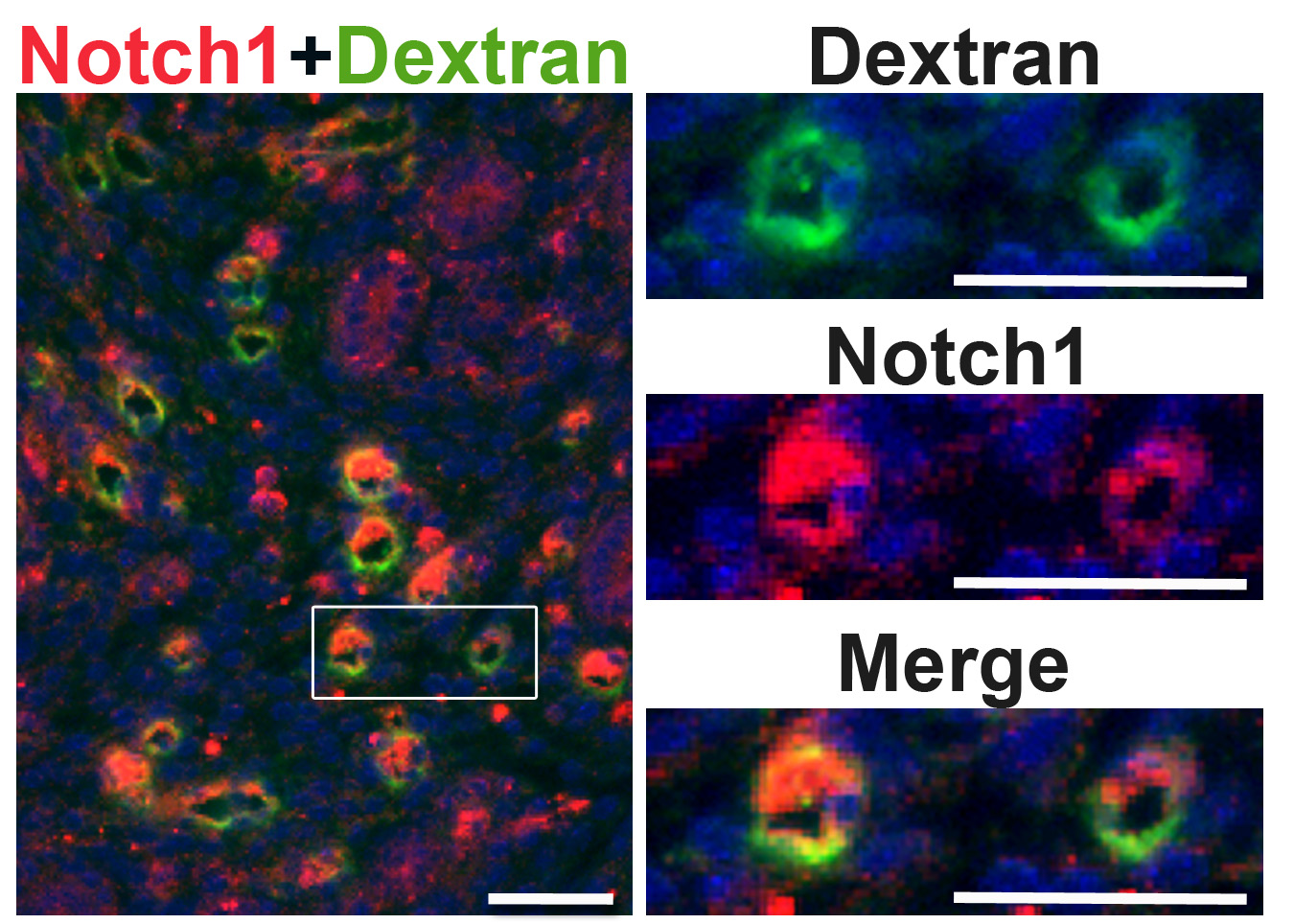

Supplement: Additional file 1: Figure S1. — Notch1 expression in patent capillaries in the pre-implantation uterus at E3.5. Notch1 expression is detected in capillaries containing FITC-dextran. The area in the rectangle is magnified and shown in panels on the right. DAPI identifies all nuclei. Scale bar = 50 μm. (JPG 373 kb) [file 13221_2015_34_MOESM1_ESM.jpg]
